# Metadherin, p50, and p65 Expression in Epithelial Ovarian Neoplasms: An Immunohistochemical Study

**DOI:** 10.1155/2014/178410

**Published:** 2014-05-22

**Authors:** Ioanna Giopanou, Vasiliki Bravou, Panagiotis Papanastasopoulos, Ioannis Lilis, Panagiotis Aroukatos, Dionysios Papachristou, Sophia Kounelis, Helen Papadaki

**Affiliations:** ^1^Department of Anatomy, School of Medicine, University of Patras, 26500 Patras, Greece; ^2^Department of Medical Oncology, Imperial College, London W6 8RF, UK; ^3^Department of Pathology, “Agios Andreas” Hospital, 26335 Patras, Greece; ^4^Department of Pathology, Elena Venizelou Hospital, 11521 Athens, Greece

## Abstract

NF-**κ**B signaling promotes cancer progression in a large number of malignancies. Metadherin, a coactivator of the NF-**κ**B transcription complex, was recently identified to regulate different signaling pathways that are closely related to cancer. We assessed the immunohistochemical expression of p50, p65, and metadherin in 30 ovarian carcinomas, 15 borderline ovarian tumours, and 31 benign ovarian cystadenomas. Ovarian carcinomas exhibited significantly higher expression of all 3 markers compared to benign ovarian tumours. Borderline ovarian tumours demonstrated significantly higher expression for all 3 markers compared to benign cystadenomas. Ovarian carcinomas demonstrated significantly higher expression of p50 and metadherin compared to borderline ovarian tumours, whereas no significant difference was noted in p65 expression between ovarian carcinomas and borderline ovarian tumours. There was a strong correlation with the expression levels of p50, p65, and metadherin, whereas no correlation was observed with either grade or stage. Strong p50, p65, and metadherin expression was associated with a high probability to distinguish ovarian carcinomas over borderline and benign ovarian tumours, as well as borderline ovarian tumours over benign ovarian neoplasms. A gradual increase in the expression of these molecules is noted when moving across the spectrum of ovarian carcinogenesis, from borderline ovarian tumours to epithelial carcinomas.

## 1. Introduction

The estimated number of new ovarian cancer cases in Europe in 2012 was 65538 with 42704 deaths [1]. Ovarian cancer is the fifth most common type of cancer in women and the fourth most common cause of cancer death in women. The majority of cases of ovarian cancer are of epithelial origin (>90%). Invasive serous carcinomas are the most common histological type accounting for up to 80% of advanced ovarian cancers. Borderline tumours comprise about 10%–15% of ovarian tumours and do not fit into the category of benign or malignant.

The NF-*κ*B family of transcription factors is expressed in many tissue types and has been studied extensively in lymphoid development and lymphoid malignancies. Constitutive NF-*κ*B signaling also has been identified in tumors of epithelial origin including breast, colon, lung, and ovarian carcinomas [2]. The NF-*κ*B transcription factor family consists of five subunits (p50, p52, p65, c-Rel, and RelB) that join into active dimers. Homo- or heterodimers form the active transcription factor complex, which is retained in the cytoplasm by Inhibitors of NF-*κ*B (I-*κ*Bs). The transcription factors are released once I-*κ*Bs are phosphorylated by I-*κ*B kinases (IKKs) upon activation by upstream stimuli. There has been previous evidence of NF-kB proteins overexpression in newly diagnosed advanced ovarian carcinomas, where a significant association of p50 with poor overall survival was found [3]. Similarly, Guo et al. demonstrated that the expression of NF-*κ*B p65 in epithelial ovarian carcinomas is mainly nuclear and that the levels correlate with poor differentiation and late FIGO stage, whereas patients who were positive for NF-*κ*B p65 subunit had inferior survival [4]. Finally, a recent study identified the network of genes controlled by the IKK*β*-NF-*κ*B pathway in one ovarian carcinoma line; using the highly specific IKK*β* small-molecule inhibitor ML120b or IKK*β* siRNA to decrease IKK*β* expression, gene expression microarray results revealed that the IKK*β*-NF-*κ*B pathway controls genes were associated with ovarian carcinoma cell proliferation, adhesion, invasion, angiogenesis, and the creation of a proinflammatory microenvironment [5]. A comprehensive review on the role of NF-*κ*B pathway on epithelial ovarian cancers can be found in reference [6].

AEG-1/MTDH (astrocyte elevated gene-1 or metadherin or lyric), originally identified as a neuropathology-associated gene in primary human fetal astrocytes [7], is now established as an oncogene in a variety of cancers, including lung [8], breast [9], diffuse large B-cell lymphoma [10], hepatocellular carcinoma [11], squamous cell carcinoma of the head and neck [12], renal cell carcinoma [13], neuroblastoma [14], prostate cancer [15], glioma [16], colorectal cancer [17], and gastric cancer [18]. Human AEG-1/MTDH gene is located in chromosome 8q22 having 12 exons/11 introns. AEG-1/MTDH is a single-pass transmembrane protein which regulates different signaling pathways that are closely related to cancer, such as nuclear factor-kappaB, Wnt/*β*-catenin, MAPK/ERK, PI3K/AKT, and AP-1 [19]. NF-*κ*B signal pathway significantly contributes to AEG-1/MTDH-mediated oncogenic events and AEG-1/MTDH activates NF-*κ*B by directly interacting with the p65 subunit of NF-*κ*B, as previously shown [20, 21]. Although AEG-1/MTDH does not directly bind to DNA, it interacts with NF-*κ*B as well as with the transcription coactivator- (CREB-) binding protein, and AEG-1/MTDH functions as a bridging factor between NF-*κ*B and CBP bringing them together to the basal transcription machinery [21].

In this study we aim to investigate the immunohistochemical expression of p50 and p65 subunits of NF-*κ*B, as well as the expression of AEG-1/MTDH in epithelial ovarian tumors: benign, borderline, and malignant, in order to further clarify their role in the pathophysiology of epithelial ovarian cancer.

## 2. Materials and Methods

### 2.1. Tissue Samples

Seventy-six ovarian neoplasms were included in our study; paraffin-embedded tissue blocks were retrieved from archival material from the Departments of Pathology of General Hospital “Agios Andreas,” Patras, Greece, and General Hospital “Elena Venizelou,” Athens, Greece. The neoplasms included 30 epithelial ovarian carcinomas, 15 borderline ovarian tumours, and 31 ovarian cystadenomas. Patients were all Caucasian and their age ranged from 12 to 81 years old, with a median age of 49. Amongst the 30 cases of carcinoma, 19 were histologically grade 3, 8 were grade 2, and 3 were grade 1. Seventeen presented with stage III disease, 6 with stage II, and 7 with stage I. All 15 borderline ovarian tumours were stage I as per FIGO, 2009 (Table 1).

### 2.2. Immunohistochemistry

Representative tumour tissue blocks were used for immunohistochemistry; 4 *μ*m thick paraffin-embedded tissue sections were deparaffinized and rehydrated. For all the antibodies used, tissue sections were subjected to heat-induced epitope antigen retrieval in 0.1 M sodium citrate using an electric microwave *ο*ven. Endogenous peroxidase activity was blocked with a 3% H_2_O_2_ solution. Protein blocking was performed with 2% bovine-serum albumin (BSA) in Tris-buffered saline (TBS). The following primary antibodies were used: anti-p50 (sc-114, 1/50, rabbit polyclonal IgG, SANTACRUZ BIOTECHNOLOGY), anti-p65 (sc-8008, 1/200, mouse monoclonal IgG, SANTACRUZ BIOTECHNOLOGY), and anti-AEG-1/MTDH (HPA015104, 1/70, rabbit polyclonal, ATLAS ANTIBODIES), overnight at 4°C. Detection of bound primary antibodies was performed using the Chem Mate TM Dako En Vision TM Detection system, Peroxidase/DAB, Rabbit Mouse (Dako, Glostrup, Denmark) according to the manufacturer's instructions. Finally, diaminobenzidine was used as a colour substrate and Harris's hematoxylin as counterstain. Appropriate positive controls (lymph nodes for p50 and p65 and small bowel for AEG-1/MTDH) were used for each antibody. For negative controls, the primary antibody was omitted. Sections from adjacent to tumour normal ovarian tissue were also stained for AEG-1/MTDH, p50, and p65 for each case included.

Immunoreactivity was scored by two pathologists and one investigator blindly to each case. The intensity of immunostaining and the distribution in the cellular population were combined in the following scoring system: 0: <10% of cells positive, 1: weak intensity in 10%–35% of cells, 2: moderate intensity in 35%–70% of cells, and 3: strong intensity in >70% of cells. Pictures were taken using the Nikon Eclipse 80i photon microscope and the Nikon DXM 1200c camera with ACT-1C software (Nikon Instruments Inc., Melville, NY, USA).

### 2.3. Statistical Analysis

The significance of differences in the expression of p50, p65, and AEG-1/MTDH (ordinal variables) between malignant, borderline, and benign epithelial ovarian neoplasms was tested using the nonparametric Mann Whitney* U*-test. Correlations between expression of proteins (immunoreactivity scores) were investigated using the Kendall Tau test. We used the binary logistic regression model to investigate the predictive value of p50, p65, and MTDH expression levels with regard to the malignant potential of epithelial ovarian tumours (dependent variable: malignant versus borderline, malignant versus benign, and borderline versus malignant). The significance level was defined as *P* < 0.05. Statistical analysis was performed with the SPSS for Windows, release 9.0 (SPSS Inc., Chicago, IL).

## 3. Results

**Figure 1:**
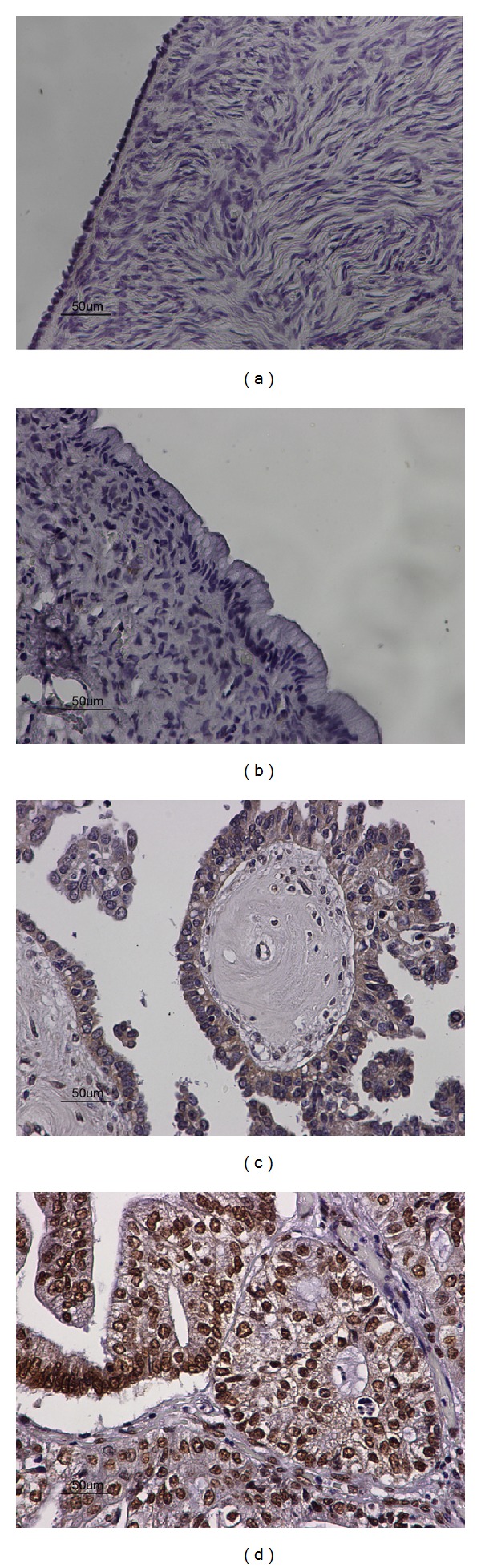
(a) Negative expression of AEG-1/MTDH in adjacent normal ovarian tissue (×40). (b) Negative expression of AEG-1/MTDH in benign ovarian cystadenoma (×40). (c) Moderate mostly cytoplasmic expression of AEG-1/MTDH in borderline ovarian tumour (×40). (d) Strong, mainly nuclear, and to a lesser degree cytoplasmic expression of AEG-1/MTDH in ovarian carcinoma (×40).

Forty-six out of our total 76 cases demonstrated high nuclear and cytoplasmic expression of AEG-1/MTDH, amongst which 30/30 carcinomas, 14/15 borderline ovarian tumours, and 1/31 benign tumours. There was no AEG-1/MTDH expression noted in normal ovarian tissue, 30/31 benign cystadenomas and 1 borderline ovarian tumour (Figures 1(a), 1(b), 1(c), and 1(d)). Ovarian carcinomas expressed statistically significant higher levels of AEG-1/MTDH protein compared to benign tumours, and borderline ovarian tumours (Mann Whitney* U*-test, *P* < 0.001). Borderline ovarian tumours demonstrated statistically significant higher expression of AEG-1/MTDH compared to the benign cystadenomas (Mann Whitney* U*-test, *P* < 0.001, Table 2). There was no statistically significant correlation of AEG-1/MTDH expression with clinicopathological parameters (stage and grade).

**Figure 2:**
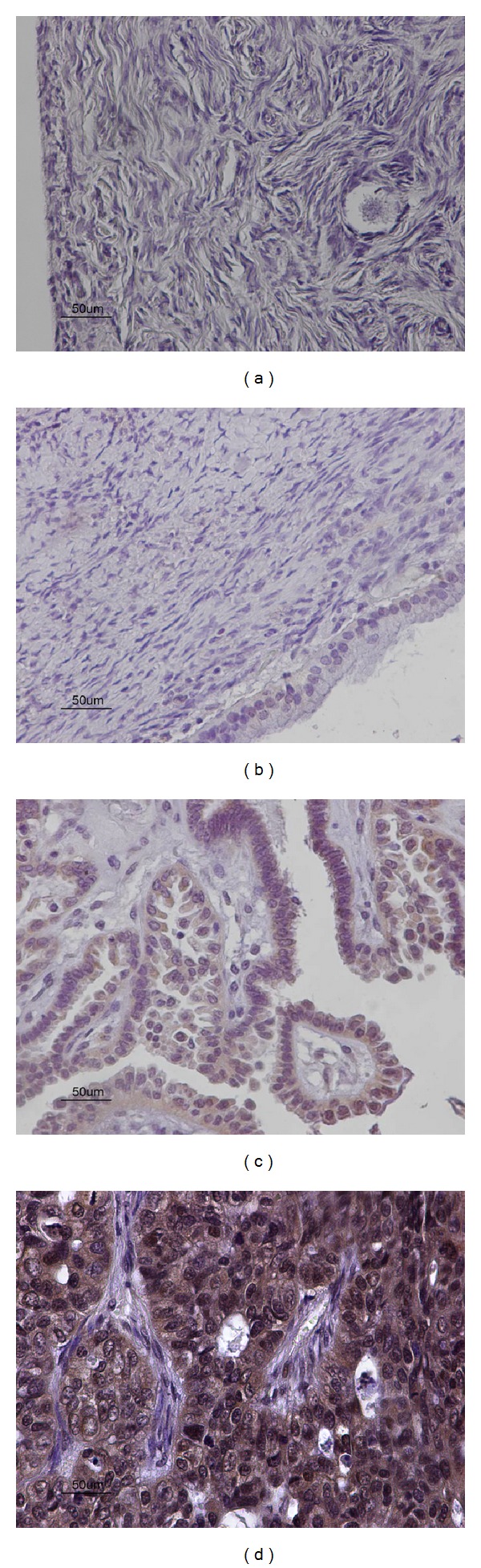
(a) Negative expression of p50 in adjacent normal ovarian tissue (×40). (b) Negative expression of p50 in benign ovarian cystadenoma (×40). (c) Moderate nuclear and cytoplasmic expression of p50 in borderline ovarian tumour (×40). (d) Strong nuclear and cytoplasmic expression of p50 in ovarian carcinoma (×40).

Forty-six out of our total 76 cases demonstrated high nuclear and cytoplasmic expression of p50, amongst which 30/30 carcinomas, 13/15 borderline ovarian tumours, and 3/31 benign cystadenomas. No p50 staining was observed in adjacent normal ovarian epithelium, 2/15 borderline ovarian tumours, and 28/31 benign cystadenomas (Figures 2(a), 2(b), 2(c), and 2(d)). Ovarian carcinomas expressed statistically significant higher levels of p50 protein compared to borderline tumours (Mann Whitney* U*-test, *P* < 0.001), and benign cystadenomas (Mann Whitney* U*-test, *P* < 0.001). Borderline ovarian tumours demonstrated statistically significant higher expression of p50 compared to the benign cystadenomas (Mann Whitney* U*-test, <0.001, Table 2). There was no statistically significant correlation of p50 expression with clinicopathological parameters of ovarian carcinomas (stage and grade).

**Figure 3:**
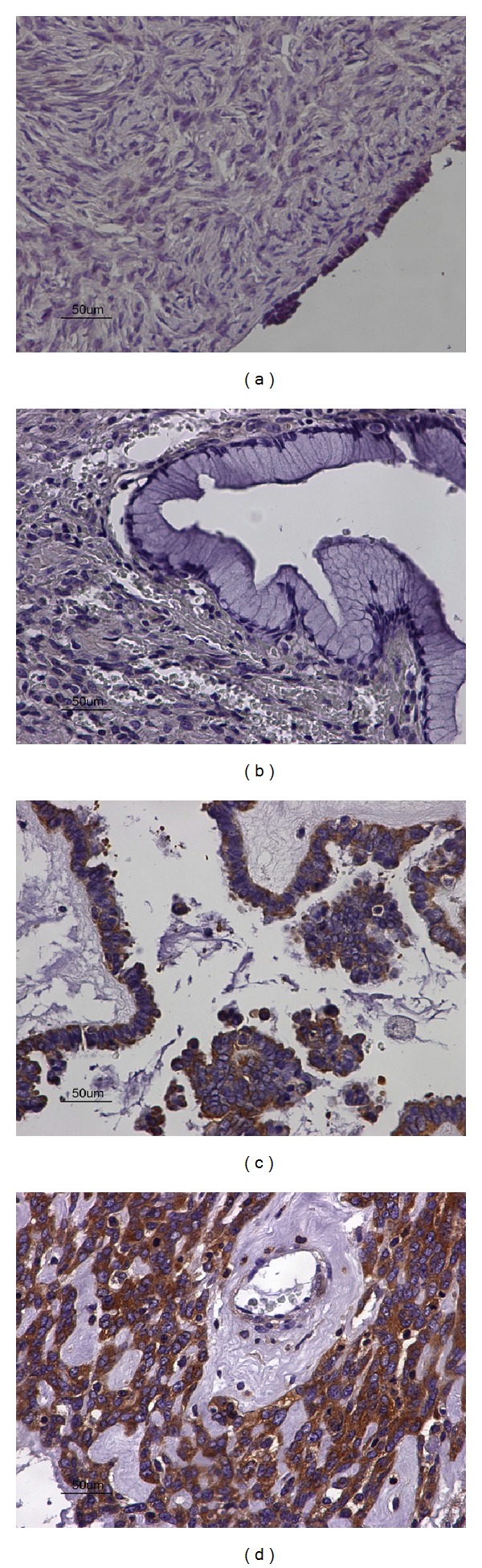
(a) Negative expression of p65 in normal adjacent ovarian tissue (×40). (b) Negative expression of p65 in benign ovarian cystadenoma (×40). (c) Moderate cytoplasmic expression of p65 in borderline ovarian tumour (×40). (d) Strong cytoplasmic expression of p65 in ovarian carcinoma (×40).

Forty-seven out of our total 76 cases demonstrated high, nuclear, and cytoplasmic (primarily), expression of p65, amongst which 30/30 carcinomas, 14/15 borderline ovarian tumours, and 3/31 benign tumours. There was no p65 expression noted in normal ovarian tissue, 28/31 benign cystadenomas, and 1/15 borderline ovarian tumours (Figures 3(a), 3(b), 3(c), and 3(d)). Ovarian carcinomas expressed statistically significant higher levels of p65 protein compared to benign tumours (Mann Whitney* U*-test, *P* < 0.001). Borderline ovarian tumours demonstrated statistically significant higher expression of p65 compared to the benign cystadenomas (Mann Whitney* U*-test, *P* < 0.001). However, there was no difference in p65 expression level between ovarian carcinomas and borderline ovarian tumours (Mann Whitney* U*-test, *P* > 0.05, Table 2). There was no statistically significant correlation of p65 expression with clinicopathological parameters (stage and grade).

Furthermore, there was a strong statistically significant correlation between AEG-1/MTDH, p50, and p65 expression levels in the 76 cases studied (Kendall Tau test, *P* < 0.001).

Finally, we used the binary logistic regression model to investigate the predictive value of p50, p65, and AEG-1/MTDH expression levels in the diagnosis of ovarian carcinomas. High AEG-1/MTDH expression was associated with a 98.4% probability of accurately distinguishing ovarian carcinomas from nonmalignant tumours. Similarly, strong expression of p50 and p65 was associated with a probability of accurate distinction of malignant epithelial ovarian tumours from nonmalignant tumours of 95.1% and 96.7%, respectively. Furthermore, high AEG-1/MTDH expression could distinguish borderline from benign ovarian tumours with an associated probability of 95.6%, whereas high p50 and p65 expression was associated with a probability of 89.1% and 91.3%, respectively. Finally, high AEG-1/MTDH expression was associated with a probability of 68.9% to accurately distinguish malignant from borderline ovarian tumours, whereas the respective probabilities for p50 and p65 were 71.1% and 68.9%.

## 4. Discussion

In this study we investigated the expression pattern of the subunits of NF-*κ*B, p50 and p65, and AEG-1/MTDH, a transcription coactivator of NF-*κ*B in ovarian carcinomas, borderline ovarian tumours, and benign cystadenomas. To our best knowledge, our study is the first report of the combined expression of AEG-1/MTDH, p50, and p65 in ovarian epithelial neoplasms indicating a potential synergistic role for the above transcription factors in epithelial ovarian carcinogenesis.

In line with our results in ovarian epithelial cancer, recently there has been emerging evidence from studies demonstrating a significant role for AEG-1/MTDH in the pathophysiology of several cancer types including breast cancer [9], large B-cell lymphoma [10], hepatocellular carcinoma, gastric cancer [11, 18], and prostate adenocarcinoma [15]. Furthermore, MTDH overexpression, inducing epithelial-mesenchymal transition, enhanced the migratory, and invasive properties of squamous head and neck cancer cells [12].

In our study, the overexpression of AEG-1/MTDH in ovarian carcinomas indicates an important role for AEG-1/MTDH in epithelial ovarian carcinogenesis and is consistent with the findings of a small number ofstudies of AEG-1/MTDH in ovarian cancer so far; AEG-1/MTDH was found to have a prognostic significance as a biomarker of distal metastasis [22], whereas high expression of AEG-1/MTDH was significantly associated with poorer overall survival and disease-free survival in another study in ovarian cancer patients [23]. Finally, Li et al. recently demonstrated that the presence of cisplatin-based chemoresistance was significantly associated with the expression level of AEG-1/MTDH in platinum-treated ovarian cancer patients [24]. In consistence with our findings, Song et al. demonstrated a gradual increase in the expression level of AEG-1/MTDH when moving from low grade benign colonic tumours (cytoplasmic expression primarily) through to frank colorectal adenocarcinoma (nuclear expression), whereas AEG-1/MTDH expression was again associated with shorter survival [17]. The cytoplasmic expression of AEG-1/MTDH observed in our study could be explained by recent evidence supporting an additional cytoplasmic role for AEG-1/MTDH in which AEG-1/MTDH serves as a binding molecule and regulator of RNA-binding proteins and components of the RNA-induced silencing complex. In the same study, depletion of cytoplasmic AEG-1/MTDH resulted in reduced cancer cell survival in response to chemotherapy [25].

In our study, p65 was primarily cytoplasmic while p50 demonstrated both cytoplasmic and nuclear localization. There have been previous studies in which high cytoplasmic p65 expression was an independent prognostic factor for biochemical recurrence in prostate adenocarcinoma and a predictor of poor survival in pancreatic cancer [26, 27]. We also acknowledge studies contradicting the significance of the cytoplasmic localization of NF-*κ*B subunits, in which p50-RelA heterodimers were found in the cytoplasm in normal and low grade cervical lesions, and subsequently translocated into the nucleus in high-grade lesions and squamous cell carcinomas [28]. Upon activation of the NF-*κ*B pathway, an upregulation of I-*κ*Bs has been described, which can potentially explain the cytoplasmic localization of NF-*κ*B subunits, as I-*κ*Bs would prevent p50 and p65 translocation into the nucleus [29]. Another possible explanation for the cytoplasmic localization of NF-*κ*B subunits is the previously identified overexpression of the nuclear membrane transporter chromosomal region maintenance/exportin1 protein (CRM1) in ovarian carcinomas [30], which has been found to facilitate the export of p65 from the nucleus into the cytoplasm.

The strong correlation in the expression levels of p50, p65, and AEG-1/MTDH further supports their significant role in epithelial ovarian cancer progression, whereas the moderate expression of the above transcription factors in borderline ovarian tumours and low expression in benign neoplasms indicate that the upregulation of NF-*κ*B pathway and AEG-1/MTDH may consist of late events in the course of epithelial ovarian carcinogenesis and further support the premalignant nature of borderline ovarian tumours. The authors acknowledge the limitations of this study; the relatively small number of cases included, along with the absence of clinical follow up data, can potentially explain the lack of correlation between p50, p65, and AEG-1/MTDH expression and clinicopathological parameters, as observed in previously mentioned studies.

Furthermore, using the binary logistic regression model we identified that high AEG-1/MTDH expression was associated with a 98.4% probability of accurately distinguishing ovarian carcinomas from nonmalignant tumours, whereas high p50 and p65 expression levels were associated with a probability of accurate distinction of malignant epithelial ovarian tumours from nonmalignant of 95.1% and 96.7%, respectively. Similar results were observed when the expression of the above markers was used to distinguish borderline from benign ovarian tumours. Our results support the potential diagnostic significance of AEG-1/MTDH, p50, and p65 expression in the differential diagnosis of ovarian carcinomas over borderline and benign epithelial ovarian tumours. Further studies on a large number of cases is required to definitively clarify the potential role of p50, p65, and AEG-1/MTDH as independent prognostic factors of the malignant potential of epithelial ovarian tumours.

## 5. Conclusion

In summary, in this study the subunits of the transcription factor NF-*κ*B, p50, and p65, and the coactivator of NF-*κ*B transcription complex, AEG-1/MTDH, were found to be overexpressed in epithelial ovarian carcinomas with a moderate expression observed in borderline ovarian tumours. We conclude that AEG-1/MTDH and NF-*κ*B signaling is active in epithelial ovarian cancer, where there appears to be a gradual increase in the expression of these molecules when moving across the spectrum of ovarian carcinogenesis, from borderline ovarian tumours to the frank malignant epithelial carcinomas. AEG-1/MTDH expression may also aid the differential diagnosis between borderline and ovarian carcinomas. Molecular techniques are required to further characterize metadherin upstream regulators and downstream mediators and investigate its potential for targeted molecular treatments.

## Figures and Tables

**Table 1:** Clinicopathologic characteristics of epithelial ovarian tumours included. (Stage as per FIGO, 2009).

	Grade 1	Grade 2	Grade 3	Stage 1	Stage 2	Stage 3
Epithelial ovarian carcinomas	3	8	19	7	6	17
Borderline ovarian tumours	n/a	n/a	n/a	15	0	0
Ovarian cystadenomas	n/a	n/a	n/a	n/a	n/a	n/a

FIGO: International Federation of Gynecology and Obstetrics.

**Table 2:** Immunohistochemical expression of p50, p65, and MTDH in epithelial ovarian carcinomas (EOC), borderline ovarian tumours (BL), and benign ovarian cystadenomas (OC). The Mann Whitney *U*-test was used for the comparison of expression levels between the 3 groups of epithelial ovarian tumours.

	p50 (nuclear and cytoplasmic)				Mann Whitney *U*-test (*P* values)	p65 (nuclear and cytoplasmic)				Mann Whitney *U*-test (*P* values)	AEG-1/MTDH (nuclear and cytoplasmic)				Mann Whitney *U*-test (*P* values)
Immunoreactivity score	0	1	2	3		0	1	2	3		0	1	2	3	

Epithelial ovarian carcinomas (EOC)	0/30	0/30	0/30	30/30	*P* < 0.001 (EOC versus BL) (EOC versus OC)	0/30	0/30	0/30	30/30	*P* < 0.001 (EOC versus OC) *P* > 0.005 (OC versus BL)	0/30	0/30	0/30	30/30	*P* < 0.001 (EOC versus BL) (EOC versus OC)

Borderline tumours (BL)	2/15	0/15	0/15	13/15	*P* < 0.001 (BL versus OC)	1/15	0/15	0/15	14/15	*P* < 0.001 (BL versus OC)	1/15	0/15	0/15	14/15	*P* < 0.001 (BL versus OC)

Ovarian cystadenomas (OC)	28/31	0/31	0/31	3/31		28/31	0/31	0/31	3/31		30/31	0/31	0/31	1/31	
